# Leg dominance influences side-specific directional movement demands during change-of-direction movements in elite youth soccer players

**DOI:** 10.3389/fspor.2026.1794849

**Published:** 2026-06-23

**Authors:** Krisztián Havanecz, Gábor Géczi, Ádám Hegedüs, János Matlák, Marcell Fridvalszki, Csaba Bartha, Sándor Sáfár, István Csáki, Péter János Tóth, Bence Kopper

**Affiliations:** 1 Training Theory and Methodology Research Center, Hungarian University of Sports Science, Budapest, Hungary; 2 Sport Management Department, Hungarian University of Sports Science, Budapest, Hungary; 3 Faculty of Kinesiology, Hungarian University of Sports Science, Budapest, Hungary; 4 Department of Sport Games, Hungarian University of Sports Science, Budapest, Hungary

**Keywords:** biomechanics, change-of-direction, IMU, leg dominance, youth soccer

## Abstract

**Introduction:**

Monitoring training load in youth soccer is a key component of athlete development. However, limited evidence exists regarding the influence of leg dominance on side-specific directional movement demands during change-of-direction movements. Therefore, the purpose of this study was to investigate the longitudinal, side-specific change-of-direction (COD) behavior and directional movement demands in Under-15 elite male soccer players, with particular emphasis on the interaction between leg dominance and movement direction during training sessions and matches.

**Methods:**

Inertial measurement unit data were collected throughout a competitive season, including direction-specific change-of-direction counts and inertial movement analysis-derived directional movement variables. Direction-specific change-of-direction frequency and inertial movement analysis-derived movement demand variables were quantified separately for dominant and non-dominant sides. Player-level mean values were analysed using mixed-design ANOVAs to examine differences between dominant and non-dominant sides and between left- and right-footed players.

**Results:**

Significant interactions were observed between side and leg dominance for inertial movement analysis-derived directional load during both trainings (*p* < 0.001) and matches (*p* = 0.003), indicating that side-specific directional load differed according to footedness rather than showing a uniform non-dominant side effect.

**Discussion:**

Consequently, side-specific directional movement demands appear to depend on the interaction between movement side and footedness, while no corresponding effects were observed for COD frequency. These findings highlight the importance of monitoring side-specific directional movement exposure and considering limb asymmetries when designing training programs in youth soccer.

## 1 Introduction

Soccer is characterized as an intermittent form of exercise, consisting of repeated short running bouts, high-intensity activity interspersed with longer periods of low- to moderate-intensity exertion (1). These physical demands include covering distance, executing rapid change-of-direction (COD), accelerations and decelerations (2, 3) with the constant activation of the lower extremity muscles. Previously, COD was thought as one of the components of agility and was defined as the ability to decelerate, change movement direction and reaccelerate in a pre-planned manner without the reaction to stimulus (4). However, nowadays the literature refers COD speed as a perceptual or decision-making component and is therefore considered distinct from agility (5). COD direction can be determined based on the orientation of the body and the direction of movement relative to a forward-facing reference (6, 7). To quantify these movement-specific variables in applied settings, wearable athlete-monitoring systems integrating global navigation satellite system/global positioning system (GNSS/GPS) technology and inertial sensors have been developed to assess players’ training load and activity profiles (8, 9).

In youth soccer, GPS-derived metrics have been used extensively to describe the physical profiles of players during both trainings and matches. Previous studies have reported that elite Under-15 players cover approximately 10 km during ∼84 min of match play, with significant age-related increases in high-speed and sprint distances (10, 11). However, GPS-derived high-speed metrics alone do not capture the full dimension of the external load, which can be broadly categorized into locomotor and mechanical demands (12). In soccer, locomotor load refers to distance- and speed-based metrics, whereas accelerometer-derived variables such as accelerations, decelerations, and COD-related measures are commonly used as field-based indicators of mechanical demands (13). Relying solely on these measures (e.g., high-speed running, sprint distance), while neglecting accelerations, decelerations, CODs and short rapid movements, may therefore provide a misleading, constrained picture of players’ physical demands (14). With the advantage of micro-electromechanical sensors, which can do inertial-based time motion analysis, there is the possibility to capture rapid micro-movement events in real-time, including high intensity accelerations, decelerations and CODs (6, 15). Wearable inertial measurement units (IMUs) have become feasible tools for detecting and quantifying COD events in applied sport settings. Validation studies report acceptable validity for detecting COD events and heading angles from IMU data, while the combined use of inertial and magnetic approaches may improve the identification of direction and angle in COD tasks (6, 7). Commercially available monitoring tools (e.g., Catapult system) now implement Inertial Movement Analysis (IMA) options that classify micro-movement events (accelerations, decelerations, left/right CODs, impacts) and report directional COD counts and magnitudes in operational dashboards.

Beyond measurement-related considerations, the interpretation of directional movement patterns also requires understanding of lower-limb dominance in soccer. It is often defined as the preferential use of one limb for skilled motor actions; however, limb dominance is also considered a multifaceted and task-specific construct that cannot be fully captured by a single definition (16). In contrast, limb preference reflects the behavioral tendency to select a limb in a given context (17, 18). Notably, these constructs are not always equivalent, as limb use may vary depending on task demands and individual characteristics, especially in team sports. Previous research suggests that laterality is not a fixed trait but may be task- and context-dependent in youth athletes (19). Importantly, limb dominance may therefore be task-specific, meaning that dominance in one motor task (e.g., kicking the ball) does not necessarily generalize to other movement actions such as change-of-direction (20).

Early match analyses in the English Premier League quantified the prevalence and positional variation of deceleration and turning movements. Also, it was found that players tend to perform on the order of 700 turns, many of which are small or moderate angles (0–90°) (21), which is one of the most common movements before the event of scoring a goal in a professional match (22). In addition, recent studies have highlighted that IMU-derived COD metrics can provide useful field-based information, although their reliability and precision may vary in youth cohorts where growth and maturation can affect movement kinematics (23–25). This further strengthens the need for longitudinal, within players approaches in COD monitoring.

Based on the points highlighted above, although previous studies have examined seasonal variations in COD performance across a competitive season (26), longitudinal on-field evidence describing direction-specific (left vs. right) trajectories of inertial COD metrics in elite youth players remains limited. This combination of side-specific inertial monitoring and longitudinal design is, to our knowledge, underrepresented in the current literature and addresses recent calls for more analyses of COD-related movement measurements. Additionally, understanding side-specific directional movement demands in relation to limb dominance may have important implications for load monitoring and non-contact injury prevention in youth soccer, as asymmetrical load patterns may contribute to uneven mechanical stress and have been associated with increased injury risk (25).

The present study therefore aims to widen current knowledge on how COD directionality relates to lower-limb dominance: (i) to examine whether left-footed soccer players exhibit a greater tendency to turn to the right, and vice versa; and (ii) to compare the differences in terms of directional movement demands in youth age groups in both trainings and matches. We hypothesize that players would perform more CODs towards their non-dominant side, irrespective of absolute movement direction (i.e., left or right), such that left-footed players are expected to turn more frequently to the right, whereas right-footed players were expected to turn more frequently to the left. We also explored whether matches were associated with greater directional movement demands than trainings. The novelty of this study lies in the longitudinal, in-season quantification of side-specific directional movement demands in trainings and matches, and in distinguishing between COD frequency and accumulated IMA-derived movement load.

## 2 Materials and methods

### 2.1 Study design

A longitudinal study was carried out during autumn and spring seasons, which included at least one official match per microcycle (27), between 31 August 2021 (first training in-season) and 21 May 2022 (last match in-season). A total of 92 training sessions (1,037 observations) and 21 matches (146 observations) were measured, with a mean duration of 64 ± 14 min of the trainings and 60 ± 8 min of the matches. Only the competitive period was considered for data collection, as the players had only recently entered the academy system and their physiological and psychological responses may have been more variable during earlier phases due to ongoing adaptation to the training and competition demands, which could have confounded the interpretation of the results. The training sessions were performed on weekdays at the same time of the day in the afternoon, at least four times a week on grass or artificial turf pitches (seasonal availability). Training sessions and matches were analysed separately, and athletes were stratified based on foot dominance (left vs. right) for independent analysis. During both training sessions and matches GPS sensors with integrated microsensors were used to obtain IMU-based data as a factor of time. Data analysis included the warm-up period, whereas the cool-down period was excluded as it consists predominantly of stretching, which contributes negligibly to training load. The training sessions were categorized using the “match day minus” (MD) format according to Malone et al., including MD-5 to MD-1 relative to the competitive match (28).

### 2.2 Participants

Twenty-one healthy elite male soccer players (*N* = 21) participated in the present study (age 14.26 ± 0.26 years, age at peak height velocity 14.05 ± 0.84 years, maturity offset 0.21 ± 0.85 years, height 168.71 ± 10.72 cm, body mass 54.47 ± 11.67 kg; all measurements are mean ± SD). Based on the participant classification framework proposed by McKay et al., the sample can be classified as Tier 3 (highly trained) (29). The players were members of a structured elite football academy system with regular training and competition. Therefore, the term “elite youth soccer players” is used to reflect their performance level within the developmental context. All the subjects were injury-free at the start of the study. Players who sustained injuries (contact, non-contact) during the observation period and were unable to fully participate in training sessions or matches were excluded from the relevant analyses from that time point onward. Additionally, data from participants who had undergone leg surgery within the past three years or during the season (actual data recording time) were excluded from further analysis (18). Goalkeepers were not considered for the study due to the significant differences in movement patterns. Notably, U15 players play 80 min in an official soccer game. For match analyses, only players who accumulated ≥60 min of total match exposure across the observation period were included, as suggested by Malamud et al. (30). Therefore, the final sample size for match analyses was 15. Players who did not meet this criterion were excluded from the match analyses only, whereas their available training-session data were retained for the training analyses where applicable. The demographic characteristics reported above refer to the full training-analysis sample (*N* = 21), unless otherwise stated.

Prior to participation, a written informed consent was obtained from all participants and their legal guardians, which included the research inclusion criteria, procedures, load, requirements, risks and benefits of the study. This research received a local institutional ethical approval (MTSE-KEB/No07/2025, 25 March 2025) for studies involving humans, and it was conducted in accordance with the latest version of the Declaration of Helsinki.

### 2.3 Limb dominance assessment

Leg dominance classification was based on self-reported kicking-leg preference, assessed using the question: “If you would shoot a ball on a target, which leg would you use to shoot the ball?” (31). The single-item question has been widely applied in laterality research, as it provides a simple and reliable measure of footedness that shows strong consistency with observed motor behavior (32). This method has been successfully applied in previous studies on the topic of unipedal postural control (33, 34); thus, we implemented this method as suggested by Promsri et al. (35). The sample comprised 11 participants with left-leg dominance and 10 participants with right-leg dominance. Therefore, in the present study, the term “leg dominance” refers to self-reported kicking-leg preference, which has been shown to provide a valid and consistent estimate of functional laterality (18), but should not be interpreted as a direct measure of task-specific functional dominance during COD actions. We also considered the influence of arm dominance, as proposed by Peters (17), as cross-dominance patterns (e.g., left arm-right leg) have been shown to influence motor control strategies, interlimb coordination, and postural regulation (36, 37). However, it is important to note that both limb dominance and limb preference are now considered task-, metric-, and context-specific constructs, which may vary depending on the movement demands (38). All participants were members of the same football academy, none were involved in other organized sports (beyond physical education classes), thereby minimizing the potential confounding influences on upper and lower limb dominance. Previous work has shown that systematic exposure to multiple sports can affect the development and expression of laterality patterns (19, 39, 40). By focusing on a single football academy and homogeneous U15 cohort, we aimed to minimize interindividual variability related to training background and enhance sample consistency.

### 2.4 Training programs

Regarding the training program, it consisted of technical, tactical, and physical conditioning development. Before each ball training session, the team's strength and conditioning coach led the warm-up session that followed the FIFA 11+ protocol standards (41). In a microcycle with only one official match, the Under-15 team trained on the second day after the previous match (MD-5). Therefore, MD-6 (the following day) was a rest day and was not included in the analyses. The weekly training structure is summarized in Table 1 according to the MD-format.

**Table 1:** Summary of the weekly training structure.

Session	Primary objective	Typical drill formats	Training load
MD-5	Offensive line-breaking, attacking organization, defensive structure	small-, medium-, and large-sided games; positional drills	Moderate load
MD-4/MD + 3	Offensive build-up, organized defense, speed and agility	speed drills, reactive drills, 1v1 to 8v8 game formats	High intensity
MD-3	Speed-endurance and match-like scenarios	small-, medium-, and large-sided games; transitions; pressing; set-piece scenarios	High intensity and volume
MD-2	Opponent-specific technical and tactical preparation	technical-tactical formats, adjusted game formats	Individually adjusted load
MD-1	Neuromuscular activation	reactive agility tasks, change-of-direction drills	Reduced load

MD-5 training days were designated as the first training day of the week, with the training objective of the development of offensive strategies for breaking through the last defensive line and completing attacking actions, while also addressing defensive mode in the other side of the field. It was extended with moderate-intensity technical work, with around 50%–60% training load compared to match situations. Also, positional drills were considered to correct issues identified in the match. The sessions incorporated small-sided possession games with constrained spaces (e.g., 6v2, 6v3 and 6v4 rondos, as well as 4v2 with two jokers across two zones). These tasks performed usually in three sets of 3-min bouts interspersed with one minute passive recovery. Medium-sided games consisted of 5v5 to 7v7 wall playing format, where players positioned “at the wall” acted as passing options while being temporarily off-field, effectively allowing passive recovery. This format included two jokers on the field, with 2 × 7, 3 × 5 and 4 × 3 min with 1.5 min rest intervals. In addition, large-sided games were conducted using wall playing format (8v8 to 10v10 with 2–4 supporting players “in the wall”) to simulate match-like attacking an defensive scenario. It consisted of 10 × 2 min loading without rest intervals (resting time was “at the wall”).

On MD-4/MD+3, the training objective was structured incorporating offensive build-up and organized defensive play on even-sided games to enhance speed and agility, including high-intensity speed drills (short sprints, agility ladder, change-of-direction), and reactive drills (e.g., coach verbal signals). This session was periodized to have approximately 90% volume of the match concerning total distance covered as the used variable. Small-sided games included changing format of 1v1 to 4v4 with small goals and the training load was set 3 × 3 min with one-minute rest intervals. The medium-sided games and large-sided games were organized in a changing format of 5v5 to 8v8, utilizing 2–4 small goals with free choice of attacking direction with 2 × 6 min loading time. The medium-sided games was conducted in a 5v5 format, employing two normal-sized goals with goalkeepers. The training load consisted of two sets of four 2-min bouts, interspersed with 2 min of passive recovery time between sets.

MD-3 targeted speed-endurance through large-sided games with and without specific constraints (e.g., free play). With the format of 8v8 plus 2 jokers with two zones with 4 goals regarding small-sided games. Considering medium-sided games, 10v8 possession was employed with 4 × 4 min work time. Large-sided games simulated match scenarios (defensive block, transitions, pressing) with match-like intensity. Defensive/offensive set-pieces in large game formats were also incorporated. The sessions took 2 × 13 to 4 × 10 min with 2–3 min rest intervals.

MD-2 emphasized technical and tactical preparations specific based on the upcoming opponent. small-sided games and medium-sided games were structured similarly to those implemented on MD-5/MD+2. The large-sided games consisted of an 8v8 tactical format conducted on half of the pitch. Typically, MD-2 was not pre-planned in terms of training load, as player exertion was continuously monitored using tracking tools till MD-3, and after that the staff members made their decision for further loading periodization. This allowed for dynamic adjustments, for example tapering strategies, which were applied by reducing individual playtime to avoid further physical stress and optimize recovery for the upcoming match.

MD-1 served as an activation day with neuromuscular focus and a reduced volume of training load. Sessions included short, high-quality neuromuscular exercises, such as activation drills, accelerations, decelerations, CODs, implemented with reaction drills. Game formats were flexible with the respect to player vs. player number, due to variations in match scheduling (e.g., early morning, midday, or late afternoon). Travel time was also taken into consideration, as it may influence players’ readiness, mood and their daily routines.

The weekly schedule included four consecutive training sessions leading into the next match. Based on this split, considering all microcycles, it ended up with 13 MD-5, 16 MD-4, 17 MD-3, 22 MD-2 and 24 MD-1. Additionally, a 30-min strength and conditioning sessions was implemented on MD-5/MD+2, MD-4/MD+3 and on MD-2 prior to ball trainings.

### 2.5 Measurement tools

IMU data was collected using a portable GPS device (Catapult Vector S7, Catapult Sports Ltd., Melbourne, Australia) integrated with tri-axial accelerometer (3D ± 16 g), magnetometer (3D ± 4,900 μT) and gyroscope (2,000 degree s^−2^) sampling at 100 Hz for every individual. The sensor was positioned vertically within the Catapult vest, located in the midline between the scapular regions. Directional movement demands were quantified using IMA-derived variables obtained from the Catapult system, representing cumulative accelerometer-based movement metrics associated with detected movement events (e.g., change-of-directions). In the present study, two conceptually distinct outcome types were analysed: COD frequency and IMA-derived directional movement load (IMA load). COD frequency was defined as the number of detected left- and right-sided COD events per session, normalized to session duration (events·min^−1^). IMA load referred to the cumulative Catapult-derived movement metric associated with detected directional movement events. COD detection and classification were based on IMU-derived data rather than GPS tracking data, allowing more accurate identification of rapid directional changes. In the present study, COD direction was operationally defined based on the manufacturer's algorithm, where left- and right-sided movement were classified according to angular ranges relative to the athlete's forward-facing orientation (0°). COD to the left was defined as movement occurring within the angular of −135° to −45°, whereas a COD to the right corresponds to movements within the 45° to 135° angular range, measured as trunk rotation around the athlete's transversal axis observed from above (42). COD direction refers to the direction of movement relative to the player's forward-facing orientation and does not indicate the stance or cutting leg responsible for force production. For clarity, directional variables were later expressed relative to individual limb dominance. Movements performed towards the dominant side are hereafter referred to as dominant (DOM), whereas movements towards the opposite side are referred to as non-dominant (NONDOM). Accordingly, the following IMA-derived variables were considered in this study: IMA CoD left low-medium-high, IMA CoD right low-medium-high, and IMA 1–12 O'Clock. The latter (IMA 1–12 O'Clock) are custom parameters that were categorized by intensity (low, medium, high) in default, but were aggregated in this study, resulting in twelve derived individual custom variables. IMA 2–4 O'Clock variables were defined as representing a COD to the right, corresponding to an angular range of 135° to 45°, while IMA 8–10 O'Clock variables represented a COD to the left with an angular range of −135° to −45°. IMA 11–1 O'Clock variables were considered as acceleration-type movements, whereas IMA 5–7 O'Clock variables were considered deceleration-type movements, which were not included in the present study. The intensity of the IMA CoD left-right variables is classified into three bands by default settings: 1.5–2.5 m s^−2^, (low-intensity), 2.5–3.5 m s^−2^ (medium-intensity), and 3.5–8.0 m s^−2^ (high-intensity). Although both COD frequency and IMA load variables are derived from directional movement events, COD frequency reflects the number of detected COD events, whereas IMA load reflects the cumulative accelerometer-derived movement demand associated with those events. Therefore, these variables should not be interpreted as interchangeable indicators. It should be noted that IMA-based directional classification relies on the proprietary Catapult algorithm; therefore, event detection and angular classification may be influenced by the device, sensor placement, algorithm settings, and movement context. Accordingly, the derived variables should be interpreted as field-based estimates of directional movement demands rather than direct biomechanical measurements.

### 2.6 Statistical analysis

The results are presented as means and standard deviations (mean ± SD). Following each physical session (trainings, matches), IMU data were collected using the OpenField Console software (Catapult Sports, Melbourne, Australia; version 3.9) and exported in.csv format for further analysis. All statistical analyses were performed using JASP software (The Jeffrey's Amazing Statistics Program; version 0.19.2, JASP Team, Amsterdam, Netherlands). Training and match data were analysed separately. COD frequency and IMA-derived directional movement load variables were normalized to session duration and expressed as events·min^−1^. Thereafter, variables were categorized relative to the dominant limb. For inferential analyses, left–right IMU variables were reclassified into dominant (DOM) and non-dominant (NONDOM) sides based on individual foot dominance. For each player, mean values were calculated across all available sessions separately for dominant and non-dominant sides, and these player-level aggregated values were used for inferential analyses. Missing sessions were not imputed; player-level means were calculated from all available valid sessions for each player. Data were aggregated at the player level across the observation period to provide stable individual side-specific profiles and to ensure that each player contributed equally to the inferential analyses, regardless of the number of recorded sessions. Mixed-effects models were not applied because the primary aim was to compare player-level side-specific patterns rather than to model session-to-session or temporal variation across the season. Each dependent variable was analysed separately using a mixed-design (repeated-measures with a between-subject factor) ANOVA, with side (dominant vs. non-dominant) specified as a within-subject factor and leg dominance (left- vs. right-footed) as a between-subject factor. Assumptions of normality and homogeneity of variance were assessed using visual inspection of Q–Q plots and Levene's tests, respectively. Effect sizes were reported as partial eta squared (*η*^2^*_p_*) and were interpreted as small (*η*^2^*_p_* = 0.01), medium (*η*^2^*_p_* = 0.06), and large (*η*^2^*_p_* ≥ 0.14). The alpha level was set at *p* < 0.05. Additionally, a chi-square test of independence was conducted to examine the association between COD direction and limb dominance based on aggregated frequency data.

## 3 Results

Table 2 presents the descriptive statistics of IMU-derived IMA COD left and right and directional movement variables collected during training sessions and matches. Mean values were generally comparable with substantial variability reflected by the SD and ranges. Higher occurrence numbers were consistently observed for directional movements performed at 3, 4 and 10 O'Clock positions in both training and match environments. No consistent differences were observed between training and match across variables, although some directional metrics showed slightly higher values in matches.

**Table 2:** Descriptive statistics of IMU-derived COD and directional variables during training and matches.

IMU-based variables	Trainings			Matches		
	mean	SD	range	mean	SD	range
IMA CoD left (low-medium-high)	168.87	64.76	378	168.35	60.31	329
IMA CoD right (low-medium-high)	180.09	59.55	355	185.85	67.60	377
IMA 2 O'Clock (low-medium-high)	24.40	10.98	68	20.99	8.19	41
IMA 3 O'Clock (low-medium-high)	60.09	28.04	169	65.68	34.56	169
IMA 4 O'Clock (low-medium-high)	82.49	30.44	172	87.97	38.22	247
IMA 8 O'Clock (low-medium-high)	20.50	8.96	63	17.25	6.74	35
IMA 9 O'Clock (low-medium-high)	38.32	15.30	96	33.39	11.70	66
IMA 10 O'Clock (low-medium-high)	80.86	36.29	239	85.00	39.26	253

IMU, inertial measurement unit; IMA, inertial movement analysis; IMA 2–10 O'Clock, movement directions categorized using a clock-face model relative to the athlete's forward-facing position; CoD, Change-of-Direction; SD, standard deviation; range, represents the minimum–maximum values observed within the sample. For the IMA CoD left/right and IMA 2–10 O'Clock variables, “low-medium-high” indicates that the default Catapult intensity bands were aggregated for the analysis. For IMA CoD left/right variables, these default bands were defined as 1.5–2.5 m s^−2^ (low), 2.5–3.5 m·s^−2^ (medium), and 3.5–8.0 m s^−2^ (high).

A chi-square test revealed no significant association between COD direction and footedness [*χ*^2^(1) = 0.02, *p* = 0.883, Cramér's *V* = 0.008].

Regarding side-specific differences, a significant side × leg dominance interaction was observed for IMA-derived directional movement load during both trainings and matches, whereas no significant effects were found for COD frequency (Table 3).

**Table 3:** Summary of the results for side-specific COD variables during trainings and matches.

Environment	Variable	Effect	*F*	*p*	*η* ^2^ *_p_*
Trainings	IMA load	Side	0.01	0.94	0.00
		Leg dominance	2.63	0.12	0.12
		Side × Leg dominance	17.41	<0.001**	0.48
	COD frequency	Side	0.09	0.77	0.01
		Leg dominance	1.99	0.18	0.10
		Side × Leg dominance	4.28	0.052	0.18
Matches	IMA load	Side	0.13	0.72	0.01
		Leg dominance	0.003	0.96	0.00
		Side × Leg dominance	13.67	0.003*	0.51
	COD frequency	Side	0.002	0.96	0.00
		Leg dominance	0.00	0.996	0.00
		Side × Leg dominance	3.68	0.077	0.22

IMA, inertial movement analysis; COD, change-of-direction; *η*^2^*_p_*, partial eta-squared. Side refers to dominant vs non-dominant side. Significant effects (*p* < 0.05) are indicated.

* *p* < 0.05.

** *p* < 0.001.

**Figure 1:**
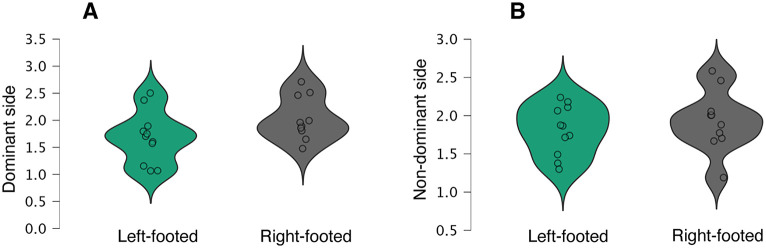
Distribution of training-related IMA-derived directional movement load according to footedness and movement side. Panel **A** shows movements performed toward the dominant side, whereas Panel **B** shows movements performed toward the non-dominant side. Individual player data are displayed to illustrate within-group variability.

As shown in Figure 1, right-footed players exhibited higher IMA load on the dominant side, whereas left-footed players showed higher values on the non-dominant side.

**Figure 2:**
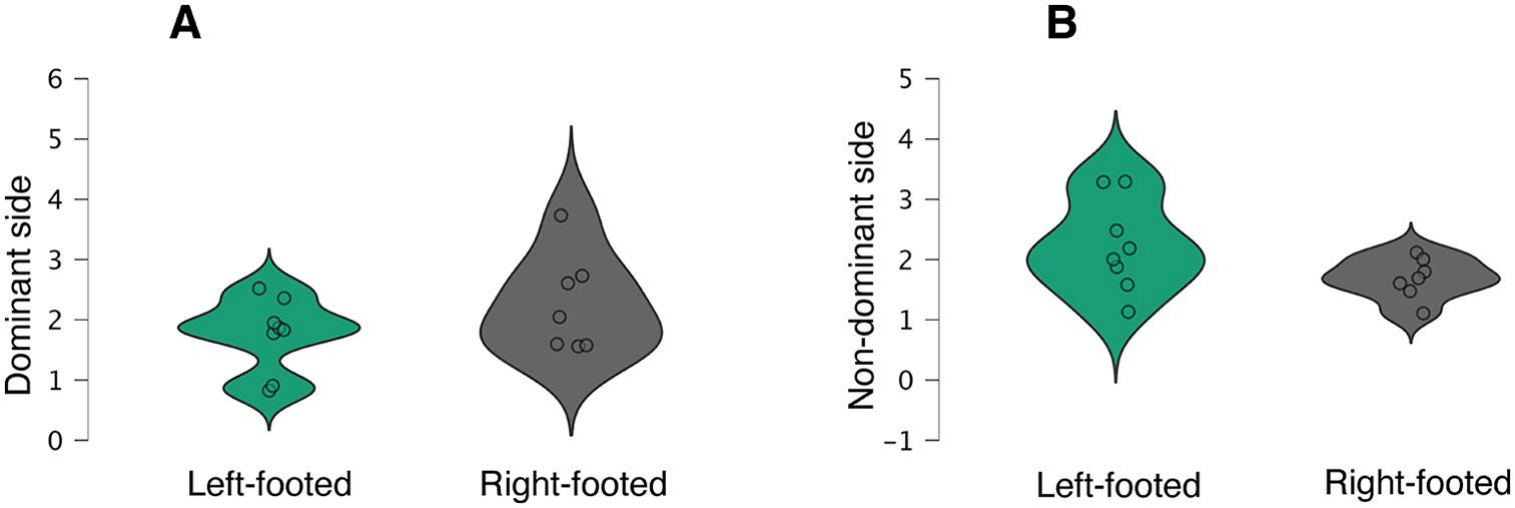
Distribution of match-related IMA-derived directional movement load according to footedness and movement side. Panel **A** shows movements performed toward the dominant side, whereas Panel **B** shows movements performed toward the non-dominant side. Individual player data are displayed to illustrate within-group variability.

A similar interaction pattern was observed during matches (Figure 2), with opposing trends between left- and right-footed players across dominant and non-dominant sides.

## 4 Discussion

The present study provides novel longitudinal evidence on side-specific COD behavior and IMA-derived directional movement load (IMA load) in U15 elite youth soccer players, with particular emphasis on the interaction between leg dominance and movement direction. The main finding of the study is the consistent presence of a significant side × leg dominance interaction for IMA load during both trainings and matches. This indicates that side-specific directional movement load differed according to footedness rather than showing a uniform dominant- or non-dominant-side effect. In contrast, COD frequency showed greater contextual variability, with no statistically significant effects in either training or match conditions. These findings suggest that while the frequency of directional changes may fluctuate depending on situational demands, IMA load reflects a more differentiated movement-demand pattern that depends on the interaction between side and footedness. It should be noted that the applied IMA-derived variables represent accelerometer-based estimates of directional movement demands rather than direct measures of mechanical load. Therefore, the present findings should be interpreted as indicators of accumulated movement exposure rather than precise biomechanical loading of the lower limbs.

The observed interaction may reflect differences in neuromuscular control and braking-reacceleration strategies between left- and right-footed players when performing side-specific directional actions, as asymmetries in braking capacity and force attenuation have been previously reported in directional movements (43–45). However, these mechanisms were not directly measured in the present study and should therefore be considered as possible explanatory factors rather than confirmed mechanisms. The interpretation is in line with the concept of the “deceleration paradox”, which highlights that higher approach velocities require greater braking forces and increase the mechanical demands during deceleration phases of movement (46). Previous research has suggested that limb dominance influences braking capacity, joint loading, and movement coordination during rapid directional changes, which may contribute to the side-specific load differences observed between left- and right-footed players (25). In youth soccer players, these effects may be further amplified by ongoing growth and maturation processes, which can transiently affect movement mechanics and inter-limb coordination (47). Consequently, the side-specific differences in IMA load identified in the present study may indicate differences in accumulated directional movement exposure that could be influenced by underlying biomechanical and neuromuscular factors, rather than differences in movement frequency alone.

In addition to biomechanical and neuromuscular explanations, external and contextual factors related to match demands and positional roles may also contribute to the observed side-specific directional movement patterns. Previous research has demonstrated that movement demands in soccer are strongly influenced by playing position and spatial occupation on the pitch, with wide winger players frequently performing high-intensity directional actions toward central areas (10, 17). Such position-specific movement profiles may partly explain the side-specific IMA load differences observed between left- and right-footed players, despite only trend-level differences in COD frequency. Moreover, match analysis studies have shown that directional actions preceding decisive events often involve rapid deceleration and reorientation towards the center of play (e.g., ball position), highlighting the importance of contextual movement direction rather than movement count alone (22). If functional leg dominance can be task- and context-dependent rather than fixed (18, 25), the interaction between limb dominance, playing position, and spatial movement demands may contribute to the side-specific directional movement load patterns observed in youth soccer players. It may be argued that more experienced or highly skilled players could exhibit more balanced directional movement behavior due to greater technical proficiency and movement adaptability.

From a methodological perspective, the use of wearable inertial sensors enabled the quantification of side-specific directional movement demands beyond traditional GPS-derived metrics. Previous studies have shown that IMU-based approaches can be used to detect COD events and estimate directional characteristics, including COD angle, in field-based settings, although the precision of these measures depends on the device, algorithm, and movement context (6–8, 48). Furthermore, although dominant leg typically emerges prior to puberty, leg use in soccer is known to be influenced by task constraints and training exposure. Experimental evidence suggests that laterality can be task-dependent and modifiable through structured practice, which may partially explain inter-individual variability in side-specific loading patterns observed in youth players (49, 50). Previous research has suggested that inter-limb asymmetries may influence physical performance and are associated with increased injury risk in athletes (51, 52), highlighting the potential relevance of monitoring side-specific movement asymmetries.

However, as direct links to performance and injury outcomes were not examined in the present study, the practical value of monitoring these variables lies in their potential to identify asymmetrical directional movement patterns relevant to performance optimization and injury risk management. Although the frequency of COD may vary according to contextual demands, side-specific differences in IMA load were observed according to footedness, suggesting that movement demand may not be fully captured by event counts alone. From a practical perspective, practitioners may consider incorporating side-specific load monitoring into training, particularly during periods of growth and maturation (e.g., adolescents), as this phase is associated with increased variability in movement coordination and a greater susceptibility to asymmetrical load patterns. Training drills that promote balance exposure to directional changes to both sides, as well as targeted neuromuscular training addressing NONDOM side control and braking capacity may help to mitigate asymmetrical movement patterns. Additionally, the use of trunk-mounted inertial sensors provides a feasible approach for longitudinal monitoring in applied settings, allowing practitioners to identify side-specific directional movement trends across training and match contexts without disrupting the training environment.

### 4.1 Limitations

Several limitations of the present study should be considered. No *a priori* sample size or statistical power calculation was performed, as the study was based on a fixed cohort of players from a single elite academy team, which constrained the sample size and did not allow for prospective determination of statistical power. The relatively small sample size limits the generalizability of the findings beyond this cohort, and the ability to detect smaller effects regarding match analyses. Therefore, the present analyses should be interpreted as being primarily sensitive to larger effects, whereas smaller or trend-level effects, especially for COD frequency outcomes, should be interpreted with caution. However, the homogeneous nature of the sample strengthens the internal validity of the observed side-specific patterns and supports the interpretation of the results within the context of elite U15 soccer players. Additionally, the higher-than-usual proportion of left-footed players (11 out of 21) in this cohort occurred by chance and may limit generalizability of the findings. It should be noted that limb dominance/preference is task-specific and may vary depending on the movement context; therefore, the classification used in this study may not fully reflect task-specific dominance during COD actions. In the present study, limb dominance was based on self-reported limb preference, which should be considered a limitation when interpreting the findings, as self-reported measures may not accurately reflect task-specific functional dominance across different movement actions. A further limitation is that the IMA-derived variables used represent field-based indicators of directional movement demands rather than direct biomechanical measures of lower-limb mechanical load. Accordingly, the findings should not be interpreted as evidence of joint loading, braking forces, or cutting-leg kinetics. Regarding biological maturation, maturity offset was not included in the statistical models, despite its potential influence on movement patterns and directional movement demands in adolescent athletes (49). Playing position was also not included as a covariate, although positional role may influence side-specific directional movement patterns. Therefore, the observed differences may be affected by inter-individual variability in maturation status, given that participants were in the adolescent age period around peak height velocity (14.05 ± 0.84 years). Although training sessions were categorized according to different training days, the analyses did not focus on specific training phases or drill-level characteristics. As a result, potential within-session variations in directional demands could not be examined in detail. Future studies may benefit from integrating drill-specific analyses to further contextualize side-specific directional movement patterns. The aggregation of data at the player level precluded the analysis of temporal changes across the season; therefore, future studies may benefit from examining within-player variability across different phases of the season (e.g., each mesocycle). Therefore, future studies with larger samples may benefit from applying mixed-effects models to examine within-player variability across different phases of the season, training contexts, or match exposure. Finally, lateral preferences in hand- and footedness typically emerge prior to puberty. However, leg use in soccer is known to be influenced by task constraints and training exposure. Previous research has demonstrated that laterality can be task-dependent and that structured practice may enhance NONDOM side involvement in youth players (49, 50). Consequently, positional demands and training design may partially modulate functional leg dominance, which should be considered when interpreting side-specific movement behavior.

### 4.2 Practical applications

From a practical perspective, the present findings highlight the importance of monitoring side-specific directional movement demands in youth soccer players, particularly in relation to leg dominance. The observed side-specific differences in IMA load, which varied according to footedness, suggest that practitioners should consider movement direction in relation to individual laterality rather than relying on movement frequency alone. Small-sided games, especially during MD-4 and MD+3 sessions, may provide an appropriate context to examine players’ directional preferences and movement-demand responses in 1v1 situations, where both physical and perceptual-cognitive demands are elevated. In practice, this may be implemented by monitoring side-specific directional movement demands during small-sided games and position-specific drills, and by deliberately manipulating task constraints to increase exposure to directional actions toward both sides. In addition, targeted neuromuscular exercises emphasizing braking control, reacceleration, and change-of-direction technique on the non-dominant side may help reduce asymmetrical movement patterns over time. Incorporating directional constraints or asymmetrical task designs may help practitioners to better understand and manage side-specific directional movement patterns, potentially supporting more balanced directional movement exposure across both sides. Monitoring side-specific asymmetries may also help identify players exposed to greater unilateral directional movement demands. Although the present study did not examine injury outcomes directly, previous literature has suggested that inter-limb asymmetries may be associated with increased injury risk in athletes (51, 52). Therefore, monitoring side-specific directional movement patterns may also be relevant from an injury risk management perspective, although this relationship should be confirmed in future studies. For practitioners without access to inertial movement unit systems, change-of-direction ability may also be assessed using field-based tests, such as the 505 COD deficit, which provides a practical alternative for evaluating directional performance (53).

## 5 Conclusion

In conclusion, this study showed that IMA-derived directional movement load during COD actions was characterized by a significant interaction between movement side and footedness in elite U15 soccer players. This indicates that side-specific directional movement demands differed according to leg dominance rather than showing a uniform non-dominant side effect. In contrast, no corresponding effects were identified for COD frequency. These findings highlight that IMA load and COD frequency represent distinct components of directional movement demands and support the use of side-specific IMU monitoring in youth soccer.

## Data Availability

The original contributions presented in the study are included in the article/Supplementary Material, further inquiries can be directed to the corresponding author.
